# ATP regulation in bioproduction

**DOI:** 10.1186/s12934-015-0390-6

**Published:** 2015-12-10

**Authors:** Kiyotaka Y. Hara, Akihiko Kondo

**Affiliations:** Department of Environmental Sciences, Graduate School of Nutritional and Environmental Sciences, University of Shizuoka, 52-1 Yada, Suruga-ku, Shizuoka, 422-8526 Japan; Department of Chemical Science and Engineering, Graduate School of Engineering, Kobe University, 1-1 Rokkodaicho, Nada-ku, Kobe, 657-8501 Japan

**Keywords:** ATP, Bioproduction, Energy metabolism, Metabolic engineering, Synthetic bioengineering

## Abstract

Adenosine-5′-triphosphate (ATP) is consumed as a biological energy source by many intracellular reactions. Thus, the intracellular ATP supply is required to maintain cellular homeostasis. The dependence on the intracellular ATP supply is a critical factor in bioproduction by cell factories. Recent studies have shown that changing the ATP supply is critical for improving product yields. In this review, we summarize the recent challenges faced by researchers engaged in the development of engineered cell factories, including the maintenance of a large ATP supply and the production of cell factories. The strategies used to enhance ATP supply are categorized as follows: addition of energy substrates, controlling pH, metabolic engineering of ATP-generating or ATP-consuming pathways, and controlling reactions of the respiratory chain. An enhanced ATP supply generated using these strategies improves target production through increases in resource uptake, cell growth, biosynthesis, export of products, and tolerance to toxic compounds.

## Background

Adenosine 5′-triphosphate (ATP) is a purine nucleotide discovered simultaneously in 1929 by Fiske and Subbarao [1] and Lohman [2]. Many metabolic reactions involve ATP synthesis and consumption. For example, 601 ATP-related reactions were listed in the KEGG database (http://www.kegg.jp) as of November 2015. ATP is required for DNA replication, biosynthesis, protein assembly, and biochemical transport (uptake and export). The role of ATP in the stress response and signal transduction is becoming rapidly defined [3–6]. Further, ATP supplies adenosine for the biosynthesis of certain metabolites.

**Fig. 1:**
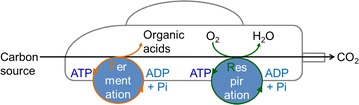
ATP generation in heterotrophic cell factories. Fermentative glycolytic and respiratory generation of ATP may be compared to the front and rear axles, respectively, of four-wheel drive vehicles

Among these roles of ATP, the energy supplies for ATP-consuming biosynthetic reactions and transport of substrates and products are important for bioproduction using cell factories [7, 8]. ATP is a universal biological energy source because of its phosphoanhydride bond, which provides a driving force to intracellular biosynthetic reactions [9]. ATP is biosynthesized by a de novo nucleotide synthetic pathway in all organisms. Many intracellular ATP-consuming enzymes utilize the biological potential energy stored in ATP (30.5 kJ/mol), and enzymatic hydrolysis of ATP generates adenosine 5′-diphosphate (ADP) and inorganic phosphate (Pi). ADP and Pi react to regenerate ATP, mainly through glycolysis in anaerobic fermentations and by the respiratory chain in aerobic bioproductions [7]. Certain acetogens synthesize ethanol from CO_2_ and H_2_ using the glycolytic and oxidative phosphorylation to generate glycolytic and respiratory ATP [10]. Thus, fermentative glycolytic and respiratory generation of ATP may be compared to the front and rear axles, respectively, of four-wheel drive vehicles (Fig. 1).

Insight into symbiosis is important in considering the generation of intracellular ATP. In eukaryotic cells, the respiratory chain resides in the mitochondrion. Mitochondrial microRNA target genes involved in energy metabolism and regulation of the ATP supply were recently identified in porcine muscle [11]. In contrast, Salvioli et al. [12] found that intracellular symbiotic bacteria regulate mitochondrial ATP generation in their host fungi and improve their host’s ecological fitness. The phosphate/oxygen (P/O) ratio, which is defined as the amount of ATP generated per molecule of oxygen consumed by mitochondria, influences growth and reproductive output, and the P/O is regulated by the generation of reactive oxygen species [13].

**Fig. 2:**
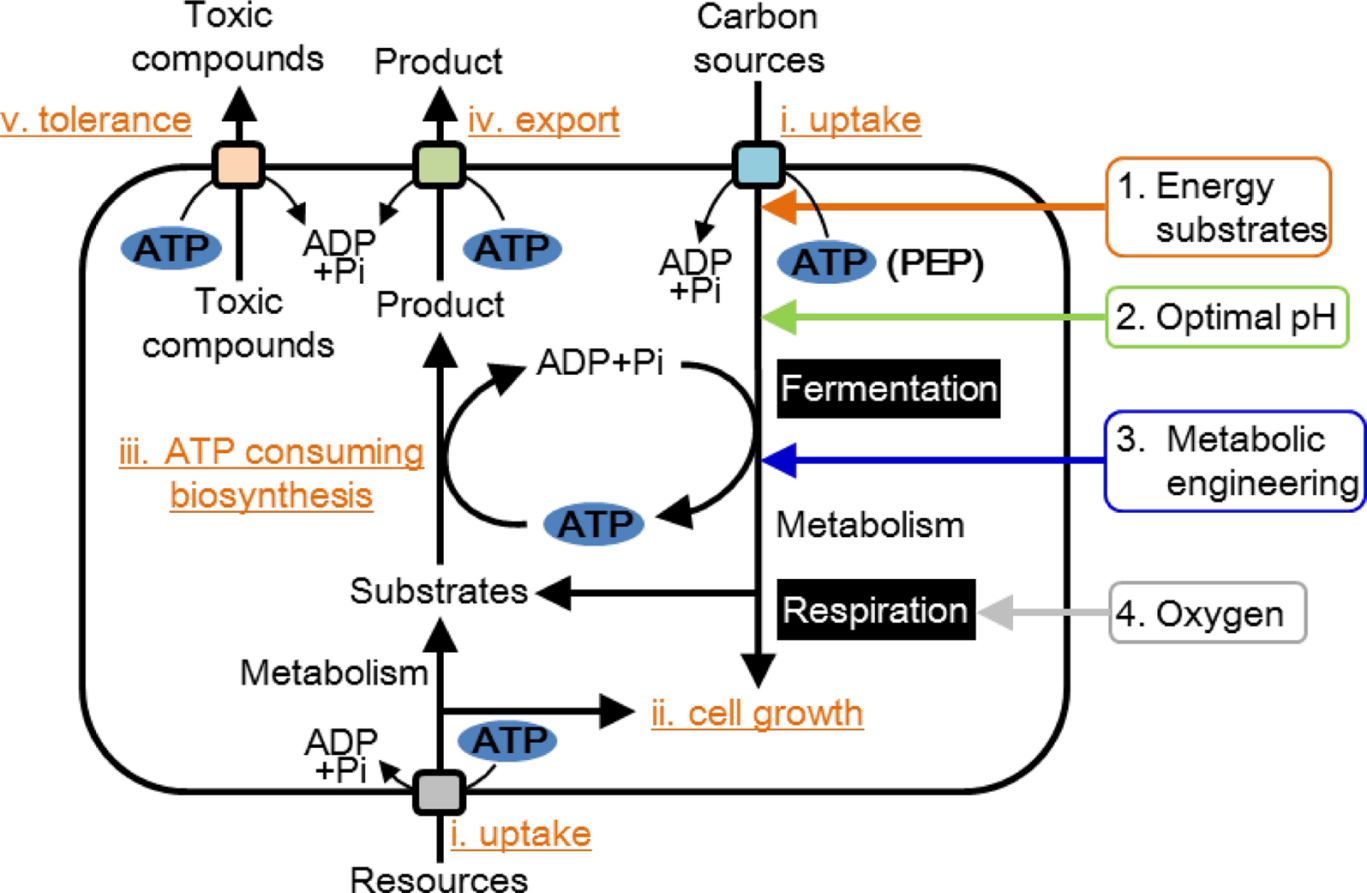
Cell factories utilize carbon source to generate ATP by glycolysis and respiratory chain. Cell factories engineered in the pathways toward target product consume much more ATP for (*i*) sugar uptake, (*ii*) cell growth, (*iii*) biosynthesis and (*iv*) export of target products, and (*v*) tolerance to toxic compounds. Cell factories improve intracellular ATP supply to drive various cellular thermodynamically unfavorable reactions with keeping high ATP supply for better bioproductions. ATP supply of the cell factories is enhanced by (*1*) addition of energy substrates, (*2*) control of pH condition, (*3*) metabolic engineering of pathways involved in ATP generation or ATP consumption and (*4*) enhancement of respiratory chain reaction

The dependence on the intracellular ATP supply (ATP generation–ATP consumption) is one of the most critical factors for bioproduction. Thus, developing cell factories with an artificially regulated ATP supply, according to a large demand for ATP, is a promising strategy to improve bioproduction yields (Fig. 2). The ATP supply is naturally regulated to maintain constant ATP levels in cells. However, the intracellular ATP supply of engineered cell factories would change because of an unnatural balance between ATP generation and consumption. Thus, improvements of the ATP supply are required to increase the production of target molecules, although it is difficult to measure the ATP supplying activity in the cell factories. For example, one of the barriers that must be overcome to achieve economical biofuel production is the enhancement of the ATP supply to maintain metabolic homeostasis of engineered cells with a higher ATP demand due to metabolic genetic engineering [14]. Metabolic simulations indicate that the maintenance of the intracellular ATP supply is a key component required to improve cell factories together with coupling cell growth and metabolic production in anaerobic and aerobic fermentations [15].

The present review focuses on current developments in regulating the ATP supply used by various engineered cell factories for improving bioproduction yields to summarize their strategies for fundamental improvement of cell factories. Four strategies to regulate the ATP supply and future perspectives will be described in the following sections. The strategies reviewed here improve resource uptake, cell growth, biosynthesis, export of target products, and tolerance to toxic compounds (Fig. 2).

### ATP regulation by energy substrates

The intracellular ATP supply is strictly regulated by a carbon source that serves as the sole energy source for heterotrophic cell factories. For example, a yeast-cell factory uses carbon sources to supply ATP required for the production of glutathione [16]. Thus, the ATP supply is very low after depletion of the carbon supply. Direct addition of ATP is critical for enhancing ATP-consuming glutathione production in *Candida utilis* after glucose depletion [17]. Exogenous addition of ATP enhances interleukin-6 production by the human epidermal keratinocyte cell line HaCaT through an increase in the phosphorylation of the epidermal growth factor receptor and the components of the p38/extracellular signal-regulated kinase pathway [18]. These results demonstrate directly that the ATP supply is rate limiting for ATP-consuming production to continue after depletion of carbon sources.

The addition of citric acid effectively increases the ATP supply. The elevated ATP supply improves the tolerance of *Candida glabrata* to extracellular pH values of 4.5–5.0 and enhances the yield of pyruvic acid [19]. Addition of citric acid as an auxiliary energy substrate for dehydrogenase reactions by malic enzyme that generate NADH enhances the contribution of electrons from NADH, which pass through the electron transfer chain to generate a proton-motive force that enhances respiratory ATP synthesis via membrane-localized F_o_F_1_-ATP synthase [19]. Citric acid addition increases the cytosolic pH and decreases the vacuolar pH. This result led to the proposal that the elevated ATP supply induced by citric acid addition enhances V-ATPase to transport H^+^ from the cytosol to the vacuole, which improves tolerance to acidic pH that is accompanied by an increase in cell growth that, in turn, increases the yield of pyruvic acid [19].

**Fig. 3:**
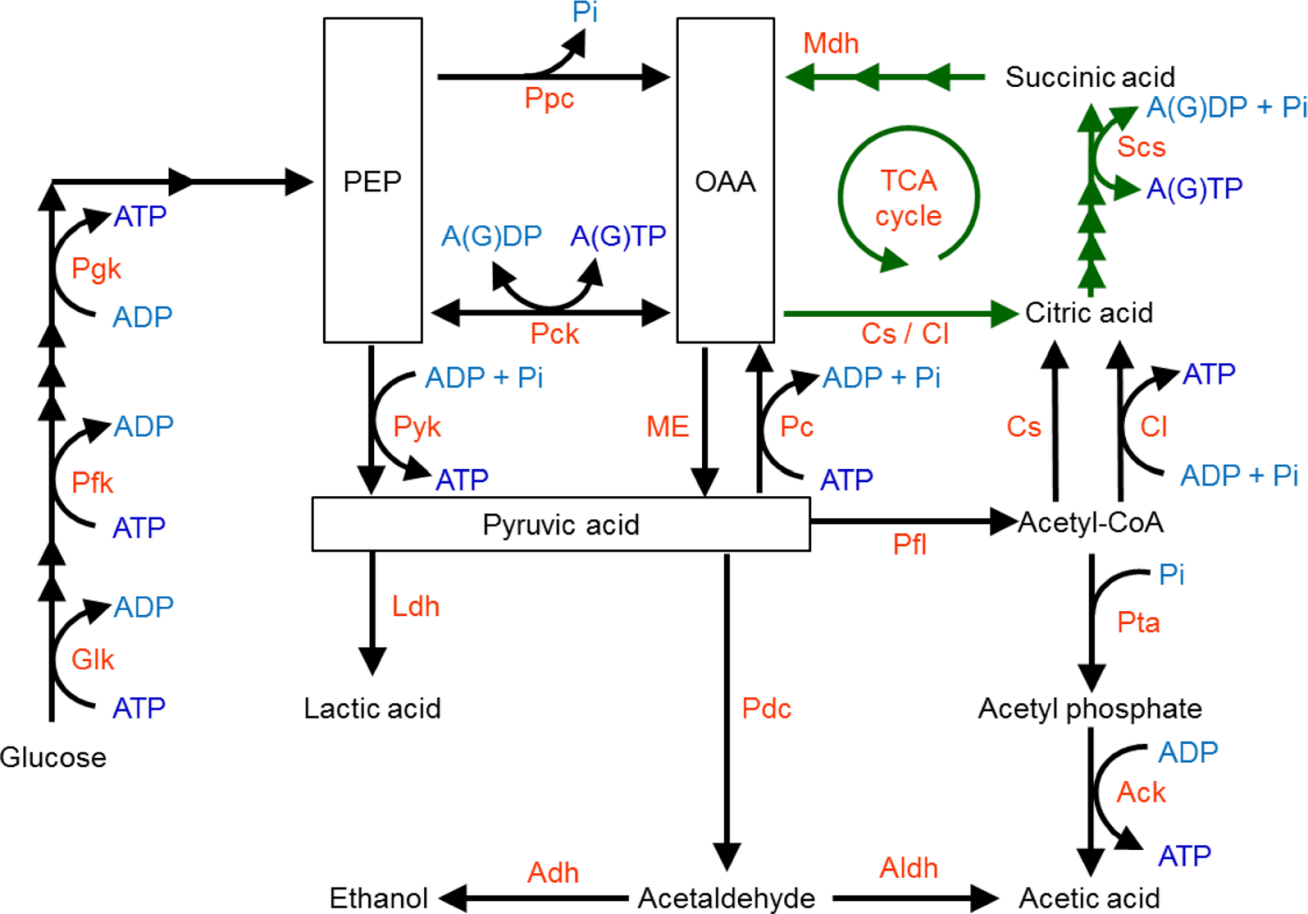
Pathways involved in ATP generation or ATP consumption. *Glk* glucokinase, *Pfk* 6-phosphofructokinase, *Pgk* phosphoglycerate kinase, *Pyk* pyruvate kinase, *Ldh* lactate dehydrogenase, *Adh* alcohol dehydrogenase, *Pdc* pyruvate decarboxylase, *Aldh* aldehyde dehydrogenase, *Pta* phosphate acetyltransferase, *Ack* acetate kinase, *Pc* pyruvate carboxylase, *Pck* PEP carboxy kinase, *Ppc* PEP carboxylase, *Pfl* pyruvate-formate lyase, *Cs* citrate synthase, *Cl* citrate lyase, *Scs* succinyl-CoA synthase, *Mdh* malate dehydrogenase, *ME* malic enzyme, *PEP* phosphoenolpyruvate, *OAA* oxaloacetate

Moreover, enhancing the ATP supply by up-regulating the expression of genes encoding citrate lyase, malate dehydrogenase, and malic enzyme, which are components of the citric acid pathway (Fig. 3), by 10- to 120-fold caused by addition of citric acid is effective for producing pyruvic acid biosynthesis in *Lactobacillus panis* [20]. During the stationary phase of growth, enhanced pyruvic acid production increases the amount of acetic acid available to generate ATP through acetate kinase. Further, enhanced pyruvic acid production increases lactic acid biosynthesis through lactate dehydrogenase (Fig. 3) and lactic acid export through a citric acid-lactic acid exchanger [20] that reduces ATP consumption required to maintain the pH in *L. panis* [20]. Overall, the increase in the ATP supply due to enhanced ATP generation and reduced ATP consumption induced by the addition of citric acid increases cell growth and lactic acid production.

These studies show that the addition of energy-generating substrates such as ATP and citric acid is critical for increasing the intracellular ATP supply. The elevated ATP supply enhances cell growth, biosynthesis, and export of target products, and improves the acid tolerance of cell factories (Fig. 2). However, using these compounds increases the total cost of industrial bioproduction.

### ATP regulation by controlling pH

Controlling pH at acidic levels enhances the intracellular ATP supply in prokaryotic cell factories, because a lower external pH confers the advantage of generating a proton-motive force between the inner and outer surfaces of the cytoplasmic membrane, which drives F_o_F_1_-ATP synthase in the respiratory chain. For example, the intracellular ATP/ADP ratio is increased in proportion to external acidity within the range of pH 3.5–4.5 under aerobic, acidic pH conditions in *Aureobasidium pullulans* [21]. Further, an enhanced ATP supply is critical for stimulating the production of pullulan, which is a linear water-soluble extracellular homopolysaccharide of glucose [21]. The strong dependency of the molecular weight of pullulan on pH shows that the increased ATP supply enhances ATP-consuming pullulan biosynthesis and may increase pullulan excretion and acid tolerance [21].

Further, the intracellular ATP supply contributes to efficient ATP-consuming peptide production under acidic conditions [22]. For example, a high influx of lactic acid into a hybridoma cell line stimulates the tricarboxylic acid (TCA) cycle and maintains malate-aspartate flux at a level that induces a high rate of ATP generation and cell growth at low pH (pH 6.8) [23]. In contrast, ATP generation and cell growth decrease at high pH (pH 7.8) owing to enhanced activity of gluconeogenic pathways [23]. Further, the ε-poly-l-lysine (ε-PL) is produced at high levels as a secondary metabolite by *Streptomyces albulus* during the stationary phase of growth. Controlling acidic pH enhances the intracellular ATP supply in *S*. *albulus*, which stimulates the enzymatic activity of ATP-consuming ε-PL synthetase [24].

The synthesis of a variety of polymers such as polysaccharides, polynucleotides, polyorganic acids, and polypeptides requires large amounts of ATP. Acidic conditions enhance the intracellular ATP supply despite increases in ATP consumption required for acid uptake to maintain cellular homeostasis. The optimal acidic conditions that exert the optimal balance between ATP generation and consumption are different in cell factories, depending on their acid tolerance. Conferring tolerance to acidic pH is a common area of interest of researchers engaged in bioproduction, because cell factories export various organic acids as byproducts. Thus, bioproduction is locked in a tradeoff between productivity and pH tolerance.

### Regulation of ATP supply by metabolic engineering of pathways that generate or consume ATP

The enhanced yields of ATP generated by the overexpression of enzymes that catalyze ATP biosynthesis are critical for increasing the ATP supply and the yields of target compounds (Fig. 2). Deletion of the gene encoding non-ATP-generating acetic acid synthetic aldehyde dehydrogenase of *Caldicellulosiruptor bescii*, which grows efficiently on biomass without conventional pretreatment, enhances ATP-generating acetic acid synthesis and increases cell growth [25] (Fig. 3). Further, deletion of the gene encoding lactate dehydrogenase of *C*. *bescii* increases cell growth owing to enhanced ATP-generating acetic acid synthesis from the carbon sources maltose and cellobiose. Combinatorial deletion of genes encoding lactate dehydrogenase and aldehyde dehydrogenase decreases the levels of lactic acid and increases the levels of acetic acid [25]. This change in the carbon flow from non-ATP-generating lactic acid synthesis to ATP-generating acetic acid synthesis increases the intracellular ATP supply. The larger pool of ATP in this engineered *C*. *bescii* strain enhances cell growth on maltose and cellobiose [25].

*S. cerevisiae* requires one molecule of ATP per molecule of ammonia to take up and assimilate the latter as a nitrogen source. In contrast, only 0.5 molecule of ATP is required for the uptake of one molecule of urea. Milne et al. [26] introduced a non-ATP consuming urease from *Schizosaccharomyces pombe* to replace the original ATP-consuming urease of *S. cerevisiae*, which confers the ability to utilize urea as sole nitrogen source. This engineered *S. cerevisiae* strain produces proteins and other nitrogenous compounds because of the availability of a sufficient supply of ATP. Heterologous overexpression of ATP-generating phosphoenolpyruvate carboxykinase (Pck) from *Actinobacillus succinogenes* in a mutant strain of *Escherichia coli* effectively enhances cell growth and succinic acid production [27] (Fig. 3). Further, succinic acid production by *Enterobacter aerogenes* is enhanced using a similar strategy that increases ATP generation by heterogeneous overexpression of Pck together with deletion of the glucose phosphotransferase system [28]. Using these engineered supplies of PEP and Pck, the PEP carboxylation pathway contributes to increase the intracellular supply of ATP [28]. Conversely, the ATP supply is insufficient to convert xylose to succinic acid, because xylose uptake requires larger amounts of ATP than the uptake of glucose [29]. An engineered *E. coli* strain lacking lactate dehydrogenase, pyruvate-formate lyase, and Pck that overexpresses ATP-generating Pck from *Bacillus subtilis* utilizes xylose and a sugarcane bagasse hydrolysate to increase succinic acid production because of an enhanced ATP supply for xylose uptake [30]. Deletion of the glucose PEP-dependent phosphotransferase system of *E. coli* increases the PEP pool, and overexpression of engineered ATP-generating Pck increases the ATP supply from this increased PEP pool and enhances succinic acid production [31, 32] (Fig. 3). Further, overexpression of ATP-generating Pck in *E. coli* increases the intracellular ATP supply during growth and enhances ATP-consuming protein biosynthesis that is dependent on the ATP supply [33]. Moreover, a significant bottleneck of recombinant protein production in yeast occurs because of ATP-consuming protein biosynthesis [34].

Cell-free systems were developed to increase the efficiency of protein production, because reaction conditions are easier to modify compared with modifying the protein synthesis machinery of whole cells [35]. Thus, cell-free protein synthesis systems are used frequently to produce proteins such as toxic and membrane proteins that are difficult to synthesize using other systems [36] and are expected to produce antibodies. Extracts of *E. coli* and wheat germ embryos are generally used for cell-free protein synthesis that depends on a sufficient ATP supply to produce the target protein [37, 38]. Therefore, cell-free systems that couple kinases to generate ATP from phosphate donors such as PEP and creatine phosphate yield a continuous supply of ATP. However, using these expensive phosphate donors increases the total cost of protein production. Thus, more efficient and economical methods for supplying ATP were developed to facilitate the use cell-free protein synthesis systems for industrial purposes. For example, a less costly method for supplying ATP was developed using the glycolytic kinases present in cell extracts in the presence of added glucose [39]. Further, combinatorial use of glycolytic kinases and creatine kinase increases the ATP supply and improves protein production [39]. Recently, the hexametaphosphate was utilized as a phosphate donor to generate ATP in a cell-free protein synthesis system [40].

Conversely, permeable (resting) cells, which are treated with detergents or organic chemicals, were developed for bio-based fine chemical production [41]. These permeable cells synthesize target products and secrete them through the permeabilized cytoplasmic membrane using less ATP compared with impermeable whole cells, which require more ATP to efflux the product (Fig. 2). In aerobic fermentation using intact whole cells, the respiratory electron transport chain supplies ATP through the proton-motive force generated between the outer and inner surfaces of the cytoplasmic membrane and the mitochondrial inner membrane in prokaryotes and eukaryotes, respectively. In contrast, permeable cells lose the ability to grow aerobically, because treatment with detergents or organic chemicals disrupts membranes, leading to the loss of ATP generation by the respiratory chain, although glycolysis continues to generate ATP [42–44]. Therefore, the ATP supply in permeable cells is usually lower compared with that of whole cells, but is remedied by coupling cellular glycolytic ATP generation with certain ATP-generating kinase reactions [45]. Further, systematic identification of genes that can be deleted to increase glycolytic ATP generation is required to enhance the ATP supply of permeable *E. coli* [46] and such deletions introduced to enhance ATP-consuming glutathione production [45]. ATP regeneration by heat-treated *E. coli* that expresses a thermotolerant polyphosphate kinase from *Thermus thermophilus* shows potential for application to ATP-driven bioproduction [47]. Conversely, another strategy to improve the glycolytic ATP supply involves inhibiting the ATP consuming glucose–glycogen bypass pathway of permeablized *S. cerevisiae* [48].

Metabolic analysis indicates that antibody production is strongly related to the intracellular ATP supply in Chinese hamster ovary (CHO) cells, which are commonly used for industrial production of recombinant proteins [49]. The intracellular production of antibodies in stationary phase is higher than during the growth of CHO cell factories. Metabolic analysis revealed that an ATP-generating Pck is more active and that an ATP/GTP-consuming Pck (Fig. 3) was less active during the stationary phase compared with the growth phase. These results indicate that a higher ATP supply in stationary phase contributes to the higher level of intracellular biosynthesis of antibodies compared with the growth phase.

In contrast, the introduction and enhancement of ATP-consuming reactions and pathways in cell factories is a strong force that drives metabolic flux in the desired direction [50]. Thus, an increase in intracellular ATP consumption stimulates ATP turnover owing to the enhancement of ATP generation and accelerates the intracellular ATP supply. For example, the butanol tolerance of *Clostridium acetobutylicum* is increased by overexpression of two ATP-consuming 6-phosphofructokinase and ATP-generating pyruvate kinase that increases the intracellular ATP supply [51] (Fig. 3). Thus, enhanced butanol tolerance is induced by the increase in the ATP supply, which is a response to the increased ATP demand from the higher ATP-turnover reactions. This strategy may improve butanol production in this engineered strain. Further, metabolic analysis of *Cyanobacteria* sp. reveals that ATP consumption by ATP through a futile cycle moderately enhances ATP turnover and increases biofuel production [52].

These studies indicate that the control of kinase reactions effectively improves ATP-consuming bioproduction by enhancing the intracellular ATP supply of cell factories.

### Regulation of ATP generation by controlling the reactions of the respiratory chain

The oxygen supply is critical for enhancing the ATP supply derived from reactions of the respiratory chain (Fig. 1). Recently, Tourmente et al. investigated the dependence of glycolysis and the respiratory chain on ATP generation by sperm [53]. They found that mice that consume higher levels of oxygen produce sperm, which depend on ATP generation by the respiratory chain rather than glycolysis, swim faster compared with those from a mouse that consumes lower levels of oxygen [53]. Moreover, an accelerated oxygen supply increases the intracellular ATP levels during lactic acid production by an engineered strain of *S. cerevisiae* that lacks the gene encoding pyruvate decarboxylase and expresses a heterologous gene encoding lactate dehydrogenase [54] (Fig. 3). The increase in oxygen supply enhances cell growth and homo-fermentative lactic acid production by this engineered strain but not by the wild-type. The ATP requirement for enhanced cell growth and lactic acid production indicates that the respiratory ATP supply is the rate-limiting factor for growth and lactic acid production of this engineered strain [54]. In *S. cerevisiae*, the relationship between the respiratory ATP supply and lactic acid production is linked by ATP-consuming lactic acid export from the cell via ATP-consuming ABC transporters [54]. Hayakawa et al. [55] compared the ^13^C-metabolic flux of *S. cerevisiae* between a parental strain and its mutant that produces higher levels of *S*-adenosyl-l-methionine (SAM). The results revealed that higher levels of SAM are produced because of an enhanced ATP supply generated by the respiratory chain, which is stimulated by the increase in TCA cycle flux [55]. Enhanced SAM production in *Pichiapastoris* is achieved by increasing the respiratory ATP supply regulated using pulsed-glycerol-feeding strategies [56]. In contrast, oxygen supply enhances intracellular ATP generation by the respiratory chain to supply ATP for ATP-consuming cellulose biosynthesis in *Thermobifida fusca*, although it inhibits cell growth [57].

Enhanced generation of ATP through the respiratory chain increases tolerance to toxic compounds. For example, alcohol toxicity is a significant problem for alcohol bioproduction. Higher ethanol concentrations produced anaerobically from pyruvic acid (Fig. 3) inhibit the activity of glycolytic enzymes. This decreases glycolytic generation of ATP and enhances ATP consumption while ethanol accumulation effectively reduces tolerance to ethanol [58]. In contrast, a butanol tolerant mutant of *S. cerevisiae* was obtained through artificial evolution under butanol stress [58]. In the final progeny, 21 of the 34 up-regulated proteins are predicted components of mitochondria, including 12 proteins of the respiratory chain [58]. These results indicate that the respiratory ATP generated by mitochondria is critical to confer butanol tolerance upon *S. cerevisiae*. Conversely, mutant *E. coli* strains lacking respiratory chain enzymes exhibit accelerated generation of glycolytic ATP and enhanced production of pyruvic and acetic acids [59] (Fig. 3). Similarly, deletion of genes encoding components of respiratory chain ATP synthase enhances the glycolytic ATP generation in permeable *E. coli* cell [44, 46] and enhances ATP-consuming glutathione production using permeable *E. coli* cell factory [45]. This enhanced glycolytic ATP generation is attributed to an increase in the expression levels of glycolytic enzymes in response to the decreased respiratory generation of ATP. Recently, Wu et al. [60] regulated the activity of the respiratory chain reaction by manipulation of the quinone synthesis pathway of *E. coli* to achieve control of lactic acid and acetic acid production.

### Future perspectives

To further improve the ATP supply of cell factories, a combination of some of strategies shown in this review may be effective. Generating multiple deletions of ATP-consuming proteins is considered a new strategy, because technology to delete multiple genes is available [61–63]. Further, deletion or overexpression of global regulators may enhance total energy metabolism. Novel strategies to increase ATP mass are critical to implement further improvements in bioproduction, such as engineering de novo ATP biosynthesis via the pentose phosphate pathway, which is accompanied by an increase in the total amounts of all adenine nucleotides. Further, an increase in other nucleotide triphosphates is critical for other specific reactions. Engineering the nucleotide synthesis pathway will be essential to control the balance of these nucleotide triphosphates.

In contrast, enhancing cell tolerance to products is strongly dependent on the intracellular ATP supply, and its enhancements represent an effective strategy to increase cellular tolerance [19, 51, 58]. Recently, biorefinery production, which is defined as bioproduction from biomass resources, is a strategy to realize sustainable industries and societies [64]. To achieve biorefinery production, pretreatment of the biomass resource is a key process, because it is difficult to use natural raw biomass materials as the direct input for cell factories. Recently, a thermostable isoamylase produced by *Sulfolobus tokodaii* was found suitable for the simultaneous gelatinization of starch and the hydrolysis of isoamylase [65]. However, most pretreated biomass materials contain chemicals that are toxic to cell factories [66]. Thus, the lack of tolerance of cell factories to these toxic chemicals is a problem for developing biorefinery production. Therefore, enhancing the ATP supply to stimulate the ability of the cell factories to export these toxic chemicals via ATP-consuming exporters is required for the future success of biorefinery production.

Measuring the intracellular ATP supply is effective for improving the output of any cell factory. Cellular ATP content is mainly measured using high-performance liquid chromatography [67] or a luciferin-luciferase assay [68, 69]. New methods are available to measure the ATP level or ATP-generating activity. For example, the intracellular ATP level is measured without extraction of ATP from cells using an ATP probe [70–72], and a modified luciferin–luciferase assay measures cellular activity that supplies ATP via glycolysis [44, 73] or the respiratory chain [74]. Using these new methods will likely be useful for improving cell factories.

Mg^2+^ is required as a cofactor for most ATP-consuming enzymatic reactions. An increase in ATP levels decreases cell growth in the presence of limiting concentrations of Mg^2+^, because Mg^2+^ is required to maintain the structural integrity of the cytoplasmic membrane [75]. Thus, sufficient supplies of Mg^2+^ and ATP are indispensable for the efficient output of cell factories.

Challenges to synthetic bioengineering approaches to enhance bioproduction, such as those outlined in this review, are rapidly increasing. Energetic cell factories using common host strains with the potential to supply high levels of ATP will likely become powerful tools to enhance diverse types of bioproduction.

## Conclusions

We focused here on the importance of the intracellular ATP supply for bioproduction. Recently, the number of studies using ATP regulation in a variety of cell factories is tended to increase. Intracellular ATP levels are normally regulated and maintained at a constant level by a robust cellular system. Indeed, in silico flux balance analysis of *Streptomyces clavuligerus* as a model organism indicates that the maximization of ATP yield is the best predictor of cellular behavior [76].

A metabolic engineering approach is very attractive for improving the cellular metabolism of the host strain to enhance the biosynthesis of target products. However, the introduction of a heterologous or the manipulation of endogenous pathways to yield the target product often consumes much more ATP than the cell can accommodate. This high ATP consumption beyond the capacity of the ATP supply disturbs the balance of ATP generation-consumption, often decreases cell growth and the saturation of end-product biosynthesis, and inhibits the export of the end-product or toxic compounds (Fig. 2). Thus, researchers employ diverse strategies to enhance the intracellular ATP supply. We categorize these strategies to regulate the ATP supply as follows: (1) adding energy substrates; (2) controlling pH; (3) metabolic engineering of pathways that generate or consume ATP; and (4) controlling reactions mediated by respiratory chain. Strategy (1) employs extracellular energy input, and its advantage is facile control of energy input by changing the amount and timing of the addition of energy substrates. However, its disadvantage is an increase in the total cost of bioproduction incurred by the addition of these substrates. Strategy (2) maintains optimal extracellular pH. Lower pH is advantageous because of its lower energetic cost for generation of the proton-motive force. The control of environmental pH can be achieved by addition of inexpensive acids. However, there is a limited pH range for enhancing the ATP supply, because lower pH inhibits either cell growth or cellular metabolism. Strategy (3) involves metabolic engineering of pathways involved in ATP generation or ATP consumption. Enhancing acetic acid biosynthesis is mainly achieved by overexpression of ATP-generating acetate kinase and deletion of lactic acid or ethanol biosynthetic pathways, or both. However, the disadvantage of this strategy is the difficulty in directing the carbon flow towards the desired pathway, because most carbon flows to the acetic acid biosynthetic pathway. In contrast, strategy (3) can be applied to the metabolic engineering of a variety of kinases. The KEGG database (http://www.kegg.jp), as of November 2015, comprised 268 kinase reactions. Strategy (4) involves metabolic engineering of the respiratory chain used mainly for aerobic bioproduction. Direct engineering of the respiratory chain is difficult because it is a large, complex system. However, the crystal structure of all of the components of respiratory complex I of *T. thermophilus* was published in 2013 [77]. Total regulation of all components based on the molecular mechanism of the respiratory chain is a subject for future studies. The strategies described here recover cell growth and overcome saturation of biosynthetic pathways by enhancing the cellular ATP supply.

## Abbreviations

ATP: adenosine-5′-triphosphate
ADP: adenosine-5′-diphosphate
SAM: *S*-adenosyl-l-methionine
PEP: phosphoenolpyruvate
TCA: tricarboxylic acid
ε-PL: ε-poly-l-lysine
